# Malignant mediastinal mesothelioma treated with anlotinib: a case report and review of the literature

**DOI:** 10.3389/fonc.2023.1153233

**Published:** 2023-05-16

**Authors:** Yishi Zhang, Wan He, Ke Zhan, Luobin Zhang, Hua Cao, Ruilian Xu

**Affiliations:** ^1^ Department of Oncology, Shenzhen People’s Hospital, The Second Clinical Medical College of Jinan University, The First Affiliated Hospital of Southern University of Science and Technology, Shenzhen, China; ^2^ Department of Spine Surgery, Shenzhen People’s Hospital, The Second Clinical Medical College of Jinan University, The First Affiliated Hospital of Southern University of Science and Technology, Shenzhen, China

**Keywords:** malignant mesothelioma, tyrosine kinase inhibitor, anlotinib, malignant pleural mesothelioma, mediastinal mesothelioma

## Abstract

Malignant mesothelioma that originates from mediastinal (MMM) is a rare form of malignant pleural mesothelioma (MPM). The prognosis of advanced stage MPM was poor, and the traditional treatment was chemotherapy. Here, we present a patient with MMM that was treated with anlotinib, a multitargeted tyrosine kinase inhibitor (TKI) who had a 24-month progression-free survival (PFS). Further review of the literature showed that, despite some explorations of applying small-molecule multitargeted TKIs in the treatment of MPM, until today, no large series had a positive result. Anlotinib had been approved by the China Food and Drug Administration on treating non–small cell lung cancer, soft tissue sarcoma, renal cell carcinoma, and medullary thyroid cancer. We assumed that the ability of anlotinib to target more tyrosine kinase receptors than most of other TKIs could contribute to the long duration of PFS in this case, but further study is needed to further validate the efficacy of anlotinib in treatment of MPM.

## Introduction

Malignant pleural mesothelioma (MPM) derives from the mesothelium, the mesoderm cell layer that lines the body cavity. Because of its mesodermal origin, the mesothelium has the potential to develop an epitheloid component and a sarcomatous component (1). Malignant mesothelioma that originates from mediastinal is rare, with limited literature reports (2). Standard treatment for advanced stage MPM includes radiation, chemotherapy, and targeted therapy; however, the treatment response is usually poor (3, 4). Here, we present a case with advanced malignant mediastinal mesothelioma (MMM) treated by anlotinib, a multitarget tyrosine kinase inhibitor (TKI), and gained long-term control of disease.

## Case report

**Figure 1:**
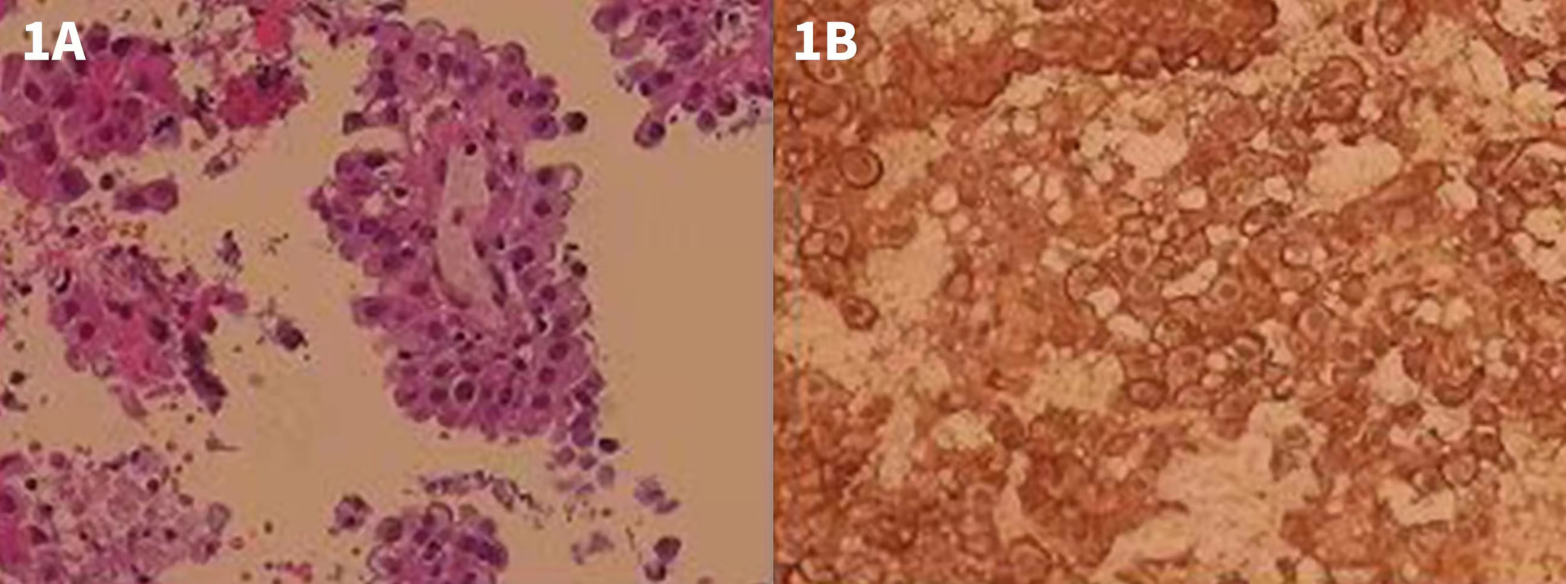
HE and IHC staining of the EBUS examination and biopsy. **(A)** HE staining showing tumor cells lined in papillary or nested pattern. Cells were with rich and eosinophilic cytoplasm as well as with heteromorphic nuclear. **(B)** IHC test showing tumor cell positive for CR.

The patient was a 65-year-old woman, complaining about coughing and hoarseness for 7 months. She experienced no other symptoms such as chest tightness, chest pain, productive cough, hemoptysis, fever, or weight loss. The patient had no history of remarkable asbestos exposure or other chronic disease. She was admitted to Shenzhen People’s Hospital on November 2017, and a plain chest computer tomography (CT) scan showed a soft tissue density mass at mediastinum. An endoscopic ultrasonography revealed a hypoechoic lesion in the mediastinum, compressing the esophagus at 30 cm from the incisor teeth. The lesion was 3.5 × 3.8 cm in size, with an irregular shape and an unclear boundary, and showed heterogeneous parenchymal echoes. On further endobronchial ultrasonography (EBUS) examination, a large area of hypoechoic lesion was detected beneath the carina. The carina widened significantly, and the left main bronchus and right middle bronchus showed stenosis due to external compressing. A fine needle biopsy was performed on the lesion, and, in histology report, the hematoxylin-eosin (HE) staining showed tumor cells lined in papillary or nested pattern. Cells were with rich and eosinophilic cytoplasm and with heteromorphic nuclear. Immunohistochemical (IHC) test showed the tumor cell positive for calretinin (CR) and human bone marrow endothelial cell marker (HBME-1), confirming the diagnosis of mesothelioma (**Figure 1**). Further abdominal CT discovered no distant organ metastasis.

From 3 January 2018 until 2 February 2018, patient received radiation: IMRT-4D GTV 64Gy/30F, concurrently with four cycles of chemotherapy with pemetrexed and cisplatin started from 9 January 2018. Imaging study revealed stable disease (SD) during the treatment and patient received regular follow-up. On October 2018, thoracic drainage of both sides was performed because of pleural effusion and malignant cells discovered in the effusion. Thus, the disease was considered progressed.

**Figure 2:**
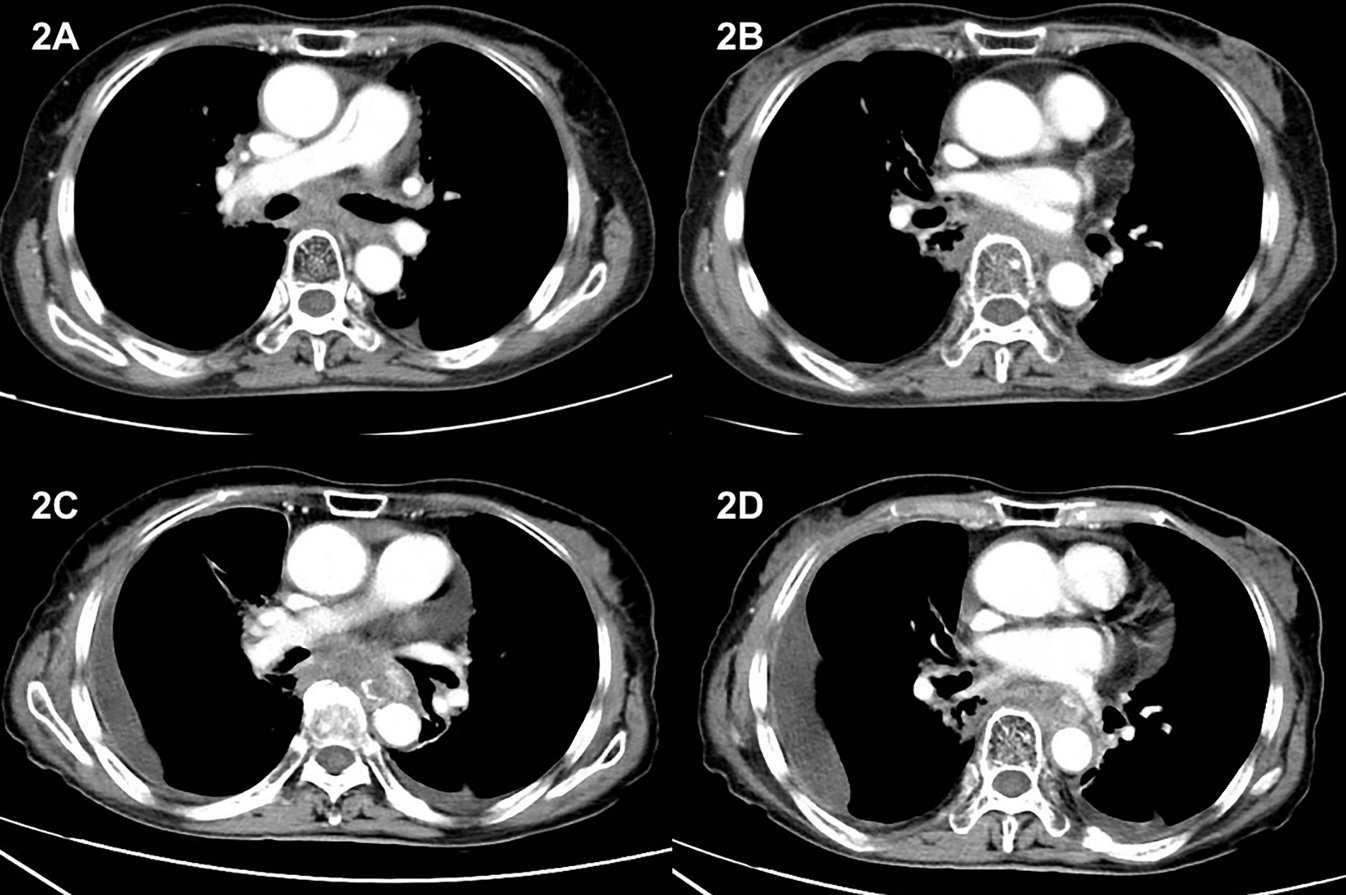
CT image during anlotinib treatment and after disease progression. **(A, B)** CT obtained on 3 March 2019, 4 months after initiating anlotinib treatment. Images showed soft tissue density mass in the posterior mediastinum and the range of the mass remained stable during anlotinib treatment. **(C, D)** CT image on 2 October 2020. Images showed enlarged posterior mediastinum mass, together with pleural effusion and pericardial effusion, indicating disease progression.

Patient refused to receive second-line chemotherapy and required for only oral medication to avoid frequently being admitted to hospital. Considering lack of other standard second-line treatment option by that time and after a detailed consultation with patient, we decided to give her an oral small-molecule tyrosine kinase receptor inhibitor as further treatment. Anlotinib, with a considerably low rate of adverse effect, was chosen as the second-line therapy. Hence, starting from 2 November 2018, she was given a single-agent anlotinib of 12 mg per os once daily for 14 consecutive days, every 21 days. Enhanced chest CT scan was performed every 3 months to evaluate treatment response, and part of the results is shown in **Figure 2**. Her best response was SD according to Response Evaluation Criteria in Solid Tumors criteria. During this treatment procedure, patient experienced level 2 blood pressure elevation according to Common Terminology Criteria for Adverse Events 5.0. After given amlodipine besylate of 5 mg/day, her blood pressure was well controlled.

**Figure 3:**
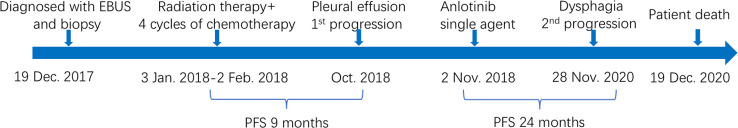
A summary of the treatment course of the patient.

On 28 November 2020, as patient began to experience symptom of dysphagia and chest CT revealed enlarged mass, progression of disease was noted. Thus, a progression-free survival (PFS) of 24 months was recorded. After the second progression, patient refused to receive further treatment other than best supportive care and died on 19 December 2020. The summary of the whole treatment course is listed in **Figure 3**.

## Discussion

MMM is a rare variant of MPM. They are considered to arise mainly from the mesothelial cells of the pericardium and comprise less than 5% of all mesotheliomas. It is sometimes referred to as “pericardial mesothelioma” in the literature (5). They may present as encasing mass involving the whole pericardium or, less frequently, as a localized tumor associated with pericardial effusion. Occasionally, invasion of the cardiac chambers and, even more rarely, metastasis to distal location have been reported (6). In our case, the tumor formed a mass at mediastinum, and pericardial effusion was presented.

The diagnosis of MMM is usually difficult because of its untypical and insidious onset (5). Sometimes, cases are discovered incidentally while patients are examined for other reason, as our case does. With disease development, hemodynamic disturbances caused by cardiac encasement and/or invasion could be noticed. Dyspnea, chest pain, weight loss, and fatigue are also common but not specific symptoms. Dysphagia only occurs in 1.4% of pleural malignant mesotheliomas, whereas its incidence rate in mediastinal mesothelioma is not clear (7). The possible mechanism might be tumor compression of the esophagus, as the barium radiography in the case that we presented showed, or, less frequently, direct infiltration of the nerves within the myenteric plexus of the esophagus, causing pseudoachalasia (8).

It was estimated that only 20% of patients with MPM are eligible for radical surgery to resect macroscopic disease (R0 or R1 resection), with a 5-year survival rate lower than 15% and a median duration of survival of 8–14 months (9). Radiotherapy also plays a key role in radical treatment, prophylactic irradiation of surgical field, and palliative cases (10). Multimodality treatment including induction chemotherapy, followed by surgery and subsequent radiotherapy, has been evaluated in several non-randomized studies, yielding a median overall survival (OS) ranging from 14 to 25.5 months (11). However, highly selected patient group may influence the outcome.

For those with unresectable MPM, traditional medical treatment is chemotherapy. Available single agent includes vinorelbine, vinblastine, mitomycin, and cisplatin, resulting in a response rate of up to 20%. Compared with the best supportive care, none of these agents could demonstrate a statistically significant benefit on survival (12). The phase III EMPHACIS trial compared cisplatin plus pemetrexed chemotherapy with single-agent cisplatin (4, 12). The median OS was 12.1 months in the double-regimen arm and 9.7 months in the control arm, demonstrating a significant survival benefit for this regimen, and also led to the Food and Drug Administration (FDA) approval of first line cisplatin plus pemetrexed chemotherapy. Other phase II–III trials explored combinations such as cisplatin plus raltitrexed, cisplatin plus gemcitabine, and carboplatin plus gemcitabine to single agent, all suggesting available options. However, taking into consideration of efficacy, toxicity profiles, and economic aspects, cisplatin plus pemetrexed still represents the first-line regimen (12).

Antiangiogenic therapies have demonstrated benefit in some patients with MPM. Angiogenesis is a complex, multifaceted process regulated by a variety of signaling process. Related signaling proteins includes vascular endothelial growth factor (VEGF), basic Fibroblast Growth Factor (bFGF), and platelet-derived growth factor (PDGF) (13). Angiogenesis is essential for tumor growth, and it has long been recognized as a therapeutic target (14). Moreover, in patients with MPM, a high microvessel density is associated with poor prognosis. Other pathways except for VEGF were also reported to be involved in MPM (15, 16). Both VEGF and fibroblast growth factor (FGF) were elevated in mesothelioma cell lines. A higher FGF-2 expression level was related to tumor aggressiveness and poor prognosis in patients with MPM. The levels of VEGF, FGF-1, and FGF-2 were also higher in mesothelioma tumor samples than that in normal mesothelium. Progranulin protein and granulin-like protein, which are VEGF-independent angiogenic factors, were also expressed in mesothelioma cell lines (17, 18). These findings support the application of multiple target inhibitors in treatment of MPM.

A phase II/III, open-label Mesothelioma Avastin Cisplatin Pemetrexed Study trial evaluated VEGF monoclonal antibody bevacizumab in combination with cisplatin/pemetrexed regimen in patients with MPM (19). In this trial, a total of 448 treatment naïve patients with unresectable MPM were randomized to cisplatin/pemetrexed with or without bevacizumab for up to six cycles. Maintenance therapy with bevacizumab was permitted, and primary endpoint was OS. Results showed that the combinational arm has significantly improved OS compared with the chemotherapy alone arm (median OS 18.8 vs. 16.1 months; HR = 0.77; 95% CI, 0.62–0.95; P = 0.0167). The HR for OS was 0.64 (95% CI. 0.40–1.02) in patients with sarcomatoid/mixed histology and 0.82 (95% CI, 0.64–1.06) in those with epitheloid histology. Thus, the triplet regimen is an option for treatment of patients with naïve, unresectable MPM.

Multiple-targeted drugs have also been evaluated. Nintedanib, a multitargeted angiokinase inhibitor targeting VEGF1, VEGF2, VEGF3, PDGFR, and FGF receptors, is approved in Europe for advanced lung adenocarcinoma after first-line chemotherapy. The phase II/III LUME-Meso trial assessed cisplatin/pemetrexed with or without nintedanib (20). In the phase II portion, the addition of nintedanib to cisplatin/pemetrexed improved PFS (median 9.4 vs. 5.7 months, P = 0.010) and was associated with a trend toward OS improvement (median 18.3 vs. 14.2, P = 0.319). However, the phase III portion of LUME-Meso trial was unable to meet its primary (PFS) and key secondary endpoints (21). Other phase I–II trials explored multiple-kinase inhibitors such as sunitinib, cediranib, sorafenib, and vatalanib in the treatment of MPM alone or in combination with chemotherapy, yet no phase III trial has been reported nor has any of these compounds been recommend by clinical guidelines (18).

Anlotinib (1-[[4-(4-fluoro-2-methyl-1H-indol-5-yloxy)-6- methoxyquinolin-7-yl]oxy] methyl) cyclopropanamine dihydrochloride) is another oral small-molecule tyrosine kinases receptor inhibitor that targets VEGF receptor, FGF receptor, PDGF receptors, and c-kit (22). It can inhibit more targets than most of other multiple-target tyrosine kinases receptor inhibitors, including sorafenib, sunitinib, and pazopanib. In preclinical studies, anlotinib has shown ability of inhibiting cell migration and formation of capillary-like tubes induced by VEGF/PDGF-BB/FGF-2 in endothelial cells. It also significantly suppressed VEGF/PDGF-BB/FGF-2–induced angiogenesis, both *in vitro* and *in vivo*. Clinical studies of anlotinib mainly focused on non–small cell lung cancer (NSCLC), soft tissue sarcoma (STS), renal cell carcinoma, and medullary thyroid cancer, and NSCLC is the most commonly studied one (22, 23).

ALTER-0303 was a multicenter, randomized, double-blind, placebo-controlled phase III trial designed to evaluate the efficacy and safety of anlotinib in patients with advanced NSCLC progressed after two or more lines of prior treatments (24). In this study, a total of 439 patients were enrolled and randomized 2:1 to receive either anlotinib or placebo, and the treatment continued until disease progression or discontinuation due to toxicity. Results showed improvements in the objective response rate (ORR) and disease control rate (DCR) in the anlotinib group compared with that in the placebo group (ORR 9.18% vs. 0.7%, P < 0.0001; DCR 80.95% vs. 37.06%, P < 0.0001). Anlotinib also significantly prolonged median PFS and OS compared with placebo (PFS 5.37 vs. 1.40 months; HR = 0.28; 95% CI, 0.19–0.31; P < 0.0001; OS, 9.63 vs. 6.30 months; HR = 0.68; 95% CI, 0.54–0.97; P < 0.0001). On the basis of above results, anlotinib was approved by the China FDA for third-line treatment or beyond in advanced NSCLC on May 2018.

A phase IIB study conducted in patients with advanced STS progressed on anthracycline-based chemotherapy compared the efficacy of anlotinib with that of placebo. As a result, the ORR and DCR in anlotinib arm were significantly higher than those in the placebo arm (ORR, 10.13% vs. 1.33%; P = 0.0145; DCR, 55.7% vs. 22.67%; P < 0.0001). In addition, anlotinib significantly improved the median PFS compared with placebo [6.27 months (95% CI, 4.30–8.40) vs. 1.47 months (95% CI, 1.43–1.57); HR = 0.33; P < 0.0001]. Another multicenter randomized phase II trial evaluated the efficacy and safety of anlotinib single agent as first-line treatment in patients with metastatic Renal Cell Carcinoma (mRCC) compared with sunitinib (25). The results showed similar PFS (11.3 vs. 11.0 months; P = 0.30), ORR (24.4% vs. 23.3%), and 6-week DCR (97.8% vs. 93.0%, P = 0.039) in the anlotinib and sunitinib groups. However, in the anlotinib group, the incidence of over grade 3 side effects (28.9% vs. 55.8%, P = 0.0039), particularly for grade 3 or 4 thrombocytopenia (0 vs. 11.6%, P = 0.003) and neutropenia (0 vs. 9.3%, P = 0.005) was lower than that in the sunitinib group.

The suggested regimen of anlotinib is 12 mg per day, administered as 2 weeks on/1 week off, and this schedule was applied in all anlotinib-related trials. In a phase I study of anlotinib in advanced refractory solid tumors, all adverse events (AEs) appeared to be manageable. The most common AEs with over 30% incidence were hand-foot skin reaction (53%), hypertension (34%), proteinuria (67%), triglyceride elevation (62%), total cholesterol elevation (62%), hypothyroidism (57%), alanine aminotransferase (ALT) elevation (48%), aspartate transaminase (AST) elevation (43%), total bilirubin elevation (38%), serum amylase (43%), myocardial enzymes abnormal (38%), leucopenia (33%), and neutropenia (33%). Although overall incidence of any AE with anlotinib was 100%, 29% reported grade 3/4 AEs, including hand-foot skin reaction (5%), hypertension (10%), triglyceride elevation (10%), and lipase elevation (5%) (22, 23). This toxicity profile agreed with those reported in other multiple kinase inhibitors such as sorafenib, sunitinib, and regorafenib. In other clinical trials, anlotinib had a similar toxicity profile.

In our case, the patient received anlotinib single-agent treatment as second line therapy after progressed on first-line radio-chemotherapy and the treatment lasted for 24 months. To date, there are no available data on anlotinib monotherapy or in combination with other therapies in treatment of MPM. Thus, this treatment decision was based on the experience of the doctors. However, given the highly malignant nature of MPM and the data of other small-molecule targeted drugs, 24 months is a praiseworthy time length. Anlotinib can target more tyrosine kinase receptors than most of other small-molecule TKIs does (23) and, together with relatively tolerable and manageable adverse effect, might explain the long duration of treatment in this case.

With appropriate biomarkers, we might be able to screen patients who might benefit from anlotinib or indicate resistance of the treatment. In fact, several biomarkers had been studied in the ALTER0303 trial (24). Activated circulating endothelial cells (aCECs) were measured in 49 patients in anlotinib arm and 30 patients in placebo arm. No statistically significant differences in baseline characteristic between both arms were found. Using cutoff of 1 for the ratio of the minimal aCEC numbers at every time point to baseline, patients in anlotinib arm were subdivided into two groups. The median PFS of the aCEC min/baseline <1 group (35 patients) was longer than that of the aCEC min/baseline >1 group (14 patients) (193 vs. 124 days; HR = 0.439; 95% CI, 0.211–0.912; P = 0.023). Therefore, aCECs could be a potential biomarker for PFS during anlotinib treatment. Circulating tumor DNA levels were also explored in ALTER 0303 trial. No correlation between sensitive EGFR mutation or EGFR T790M mutation and PFS was discovered. However, more evidence is needed to further validate the role of biomarkers in anlotinib treatment.

Immune checkpoint inhibitors (ICIs) offered new expectation in MPM treatment. In addition, 16% to 68% patients with MPM had a positive programmed cell death ligand 1 (PD-L1) expression, which might partially explain ICI’s potential efficacy. Among the corelative trials, the combinational application of cytotoxic T-lymphocyte–associated protein 4 (CTLA-4) inhibitor and PD-1 inhibitor achieved yet best efficacy. In Checkmate-743 trial, an open-label, multicenter, randomized phase III clinical study designed to compare the efficacy and safety of nivolumab in combination with Ipilimumab versus first-line chemotherapy in patients with advanced-stage MPM, 605 patients were recruited (26). As a result, the ICI arm had an OS of 18.1 months, compared with 14.1 months in chemotherapy arm (HR = 0.74, P = 0.002). The 1-year OS rate was 68% versus 58%, and the 2-year OS rate was 41% versus 27%. On the basis of this result, nivolumab in combination with ipilimumab was recommend as the first ICI regimen by the National Comprehensive Cancer Network (NCCN) guideline for first-line treatment in advanced MPM. Pembrolizumab, another PD-1 inhibitor and single-agent Nivolumab, also showed certain efficacy in patients with advanced-stage MPM after standard chemotherapy in Keynote-028 study and NivoMes study (27, 28), respectively. Nevertheless, most ICIs achieved limited duration in treatment response in MPM while further study on this field is under development.

In conclusion, we reported a relatively rare case of MMM. The patient had a 24-month disease control with treatment of a multiple-targeted medicine anlotinib, and adverse effect during the treatment was controllable. This case indicated that anlotinib could be a potential treatment option for management of malignant mesothelioma.

## Data availability statement

The original contributions presented in the study are included in the article/supplementary material. Further inquiries can be directed to the corresponding author.

## Ethics statement

Written informed consent from the patients was not required to participate in this study in accordance with the national legislation and the institutional requirements.

## Author contributions

YZ, WH, HC and RX had full access to the data and took final responsibility for the decision to submit for publication. All authors contributed to the article and approved the submitted version.
